# Healthcare exceptionalism: should healthcare be treated differently when it comes to reducing greenhouse gas emissions?

**DOI:** 10.1007/s11019-025-10254-x

**Published:** 2025-01-25

**Authors:** Joshua Parker

**Affiliations:** Faculty of Health and Medicine, Health Innovation One, Sir John Fisher Drive, Lancaster University, Lancaster, LA1 4AT England

**Keywords:** Climate change, Distributive justice, Cliamte justice, Exceptionalism, Sustainability in healthcare, Ability to pay principle

## Abstract

Healthcare systems produce significant greenhouse gas emissions, raising an important question: should healthcare be treated like any other polluter when it comes to reducing its emissions, or is healthcare special because of its essential societal role? On one hand, reducing emissions is critical to combat climate change. On the other, healthcare depends on emissions to deliver vital services. The resulting tension surrounds an idea of healthcare exceptionalism and leads to the question I consider in this paper: to what extent (if any) should the valuable goals of healthcare form an exception to the burdens of reducing greenhouse gas emissions? The goals of this paper are twofold. One is to think about how to address the issue of healthcare exceptionalism. Second is to discuss the extent of healthcare’s climatic responsibilities. I examine two perspectives on healthcare exceptionalism. The first treats a responsibility to reduce emissions and the delivery of healthcare as separate issues, each governed by its own principle. I reject this view, proposing instead that we consider healthcare’s environmental responsibilities in conjunction with its essential functions. I defend an “inability to pay” principle, suggesting that while healthcare should indeed contribute to mitigating climate change, its obligations should be constrained by the necessity of maintaining its core goals like protecting health and preventing disease. Healthcare should be treated differently from other sectors, but not to the extent that it is entirely exempt from efforts to reduce emissions.

## Introduction

A tension results from our intuitions about the importance of tackling climate change and how this affects institutions we think of as special, like healthcare. Healthcare is a significant source of greenhouse gas emissions and in the face of climate breakdown healthcare emissions should be reduced. And yet, if healthcare is special because of its role in protecting goods like health, then this offers a justification for thinking of healthcare differently in the allocation of mitigation responsibilities. So, is there something special about healthcare that means that when it comes to tackling climate change, healthcare does not have the same responsibilities as other polluters like air travel or fashion?

The issue of how complex and technologically advanced healthcare systems reconcile providing the benefits of healthcare with the challenge of minimising the emissions they have historically relied on to provide those benefits is central to questions of healthcare’s climatic responsibilities. This paper is concerned with how to resolve this tension. The question I raise is one of exceptionalism: to what extent (if any) should healthcare be treated as exceptional when it comes to mitigation burdens?

At its core, this question examines whether healthcare is, or should be, considered special and thereby treated as distinct from other sectors, particularly when it comes to climatic responsibilities. Healthcare exceptionalism suggests that there are ways in which we take healthcare to be an exception to general rules or obligations. Take the idea that polluters should pay in proportion to their emissions, healthcare exceptionalism would object to a general principle of treating all polluters alike due to healthcare’s perceived importance. In the context of climate policy, this concept implies that healthcare should be exempt from certain duties, such as reducing its emissions, due to healthcare’s vital role in safeguarding and promoting health.

My goals in this paper are twofold. One objective is to offer a framework for thinking about the issue of healthcare exceptionalism in mitigation responsibilities. Mitigation refers to actions that limit the impact of emissions on climate change either by preventing emissions or enhancing activities that remove greenhouse gases from the atmosphere. Healthcare exceptionalism is a scalar concept as healthcare could be treated more or less differently in the allocation of mitigation burdens. Where one falls on this spectrum influences what is considered healthcare’s fair share of mitigation burdens. To articulate this spectrum I use Caney’s distinction between ‘isolationism’ and ‘integrationism’ (Caney 2012, 2018). This distinction relates to whether mitigation responsibilities and providing the benefits of healthcare should be treated as separate issues, or integrated. Isolationism treats mitigation responsibilities and healthcare benefits as separate issues, while integrationism combines them. In this paper I reject isolationist positions and sketch a view that integrates concerns about climate change with meeting the goals of healthcare.

My second goal is to argue for a moderate position on this spectrum. I propose that healthcare’s mitigation responsibilities should be determined based on ability to pay, or more precisely on when healthcare has an *in*ability to pay. Healthcare is liable to mitigate its emissions unless doing so would threaten its ability to satisfy basic needs. This means some emissions are morally permissible and leaves a sphere of healthcare emissions that are treated differently, but not all healthcare emissions are exempt.

The paper is structured into three main sections. The first section is concerned with clarifying the nature of the problem and furnishing the distinction between isolationism and integrationism. This is fundamental to how I resolve the issue of healthcare exceptionalism. The first section also clarifies what I mean when I refer to ‘healthcare’ as having certain responsibilities. In the second section I discuss two approaches that compartmentalise the goals of healthcare and mitigation responsibilities: ‘healthcare non-exceptionalism’ and ‘absolute healthcare exceptionalism’. The first view, ‘healthcare non-exceptionalism’ rejects the idea that healthcare is special and disregards the goals of healthcare focusing just on mitigation responsibilities. I argue against this and turn to examine whether treating healthcare as special means we should exempt it from mitigation responsibilities. The final section of the paper concerns how to reconcile the goals of healthcare with mitigation responsibilities. It is here that I make the case for an inability to pay principle. Using ability to pay, it is possible to delineate some exceptions to mitigation for healthcare on the basis that healthcare is sometimes necessary to secure individual’s basic needs but emissions beyond this are liable to mitigation. Healthcare should therefore be treated differently from other sectors, but not to the extent that it is entirely exempt from efforts to reduce emissions.

## Preliminaries

To begin, I clarify the nature of the problem before explaining the method by which exceptionalism can help frame the conflict between these two important goals.^1^

Healthcare accounts for 4–5% of emissions globally (Health Care Without Harm 2019; Lenzen et al. 2020). Healthcare emissions in different countries account for a greater or lesser proportion of national emissions. The National Health Service (NHS), for example, makes up 4% of emissions in England. This equated to 25 megatonnes of CO_2_ equivalent in 2019 (Tennison et al. 2021). Compare this to healthcare in the United States where healthcare emissions are closer to 10% of national emissions. (Eckelman and Sherman 2016) The threats posed by climate change and the need to stay within climatic targets creates a strong impetus to reduce emissions. As healthcare has a significant carbon footprint, we may think a responsibility to mitigate emissions extends to healthcare.

When discussing healthcare’s climatic responsibilities, it is important to be clear on what I mean by ‘healthcare’. I use ‘healthcare’ and ‘healthcare system’ interchangeably to refer to the organised efforts of societies to promote health, prevent disease and provide medical care. Despite different funding models and structures worldwide, healthcare systems share the common goals of promoting and protecting health, alleviating symptoms, preventing premature death, and providing end-of-life care (Brülde 2001; Hastings 1996; Pellegrino 2001; Schramme 2017).

Healthcare systems are complex as a result of the increasing complexity of the problems they address, the array of technologies and methods of addressing healthcare problems and the numerous people—politicians, managers, healthcare professionals and staff—who play roles in organising and delivering care. When I refer to ‘healthcare,’ I’m referring to this group of individuals responsible for ensuring the system functions effectively and fulfils its core purposes. These individuals are the main duty-bearers responsible for reducing healthcare’s emissions.

I do not go so far as to describe healthcare as a collective agent with moral responsibilities beyond those of its members (Smiley 2023), rather I view it as a group of individuals with shared responsibilities. Therefore, when I use the term ‘healthcare,’ it should be understood as shorthand for the group of people responsible for making sure healthcare systems can function and meet their goals. Clearly there is a further question of how to allocate responsibilities amongst these various actors, but in this paper I am interested in the responsibilities healthcare has regarding climate change.

There is no canonical blueprint for a low-carbon or net-zero healthcare system. Nonetheless, so far 81 healthcare systems around the world have committed to become sustainable and low carbon, and 45 have committed to net-zero (World Health Organisation 2024). It is difficult to specify precisely what actions healthcare systems can and should take to reduce their emissions where there are commitments without fully worked out plans for sustainable healthcare systems. To make a start on reducing healthcare emissions, it is important to first appreciate the makeup of healthcare’s carbon footprint. To use the English NHS as an exemplar, one study found that the supply chain which includes medicines, equipment and the like, account for most of its greenhouse gas emissions (62%) (Tennison et al. 2021). Direct patient care results in 24% of emissions and the remaining carbon footprint is from patient and staff travel, and commissioned services.

Driving healthcare emissions down means targeting the sources of emissions cited. At the most general level, reducing the carbon footprint of healthcare is thought to entail changing what, where and how healthcare is provided (Naylor and Appleby 2012). A comprehensive and wide-ranging shift in how healthcare is structured, organised and delivered is expected to be required to decarbonise healthcare (National Health Service 2020). This includes, but is not limited to: creating a culture of sustainability, tracking and reporting the carbon footprint of healthcare, offering financial incentives to reduce emissions, green supply chain sourcing, shifts in energy use including renewables and energy conservation, low carbon transportation, low carbon foods and packaging as well as minimising waste, prioritising disease prevention and chronic disease management, and, reducing overtreatment and overprescribing (Salas et al. 2020). Investments in infrastructure and low-carbon technologies, as well as shifts in how and what healthcare is offered, can lead to opportunity costs, especially in the shorter term, as funds spent on mitigation are diverted away from direct patient care. But more fundamentally, the extent to which healthcare should change, the burdens it should shoulder in decarbonising and what the resulting healthcare system looks like all depend on the principles of justice we adopt in guiding the transition to lower carbon healthcare.

As mitigation is burdensome and involves a transition in the structure, organisation, and perhaps even the function of healthcare, healthcare systems that seek to minimise their emissions will be quite different to ones with no such commitments. There are two goals to consider here. The goals in question are (1) minimising the threats of climate change through mitigation, and (2) the ends of healthcare like treating disease, minimising suffering, protecting health and so forth. As each goal shapes what decisions are made and what constraints are placed on healthcare, how each goal is adopted, implemented, and constrains and disrupts the other sculpts healthcare systems and consequently the distribution of benefits and burdens within it. Put another way, policy makers, managers and clinicians will make quite different decisions if their primary goal is to reduce emissions, to promote the health of certain populations, or both. When stakeholders make decisions on this basis, the nature of healthcare systems, and in turn the distribution of certain goods like health in a population, alter. The issue at stake is one of distributive justice– the fair distribution of the burdens of climate change mitigation and the benefits of healthcare– and is essential in understanding what healthcare systems should do when it comes to climate change.

Healthcare exceptionalism enters the debate as a response to the idea that healthcare should carry the burdens of reducing its emissions. Some may object that imposing a green agenda on healthcare is unfair. Policymakers may worry that environmental goals could negatively impact the delivery of care, while doctors might see climate change as unrelated to their duty to treat patients. Patients may also resist efforts to reduce emissions if they feel it compromises their healthcare entitlements. These reasonable concerns ultimately stem from the belief that healthcare is special and should be treated differently.^2^ Even those who support reducing healthcare emissions may argue that there should be limits on the extent of these efforts, based on the idea of healthcare exceptionalism and concerns about fairness.

There are two methods for resolving the conflict between healthcare’s overarching goal to protect health and mitigation burdens: an isolationist method and integrationist one. Isolationism is the idea that principles of justice should focus on just one good and be applied in isolation from wider considerations. Integrationism, on the other hand, applies a general principle of justice to a whole package of goods, considering them as a whole (Caney 2012, 2018). The methodological distinction between isolationism and integrationism helps us understand the different ways to approach the conflict between the goals of healthcare and the demands of climate change mitigation. The first method views each goal as a stand-alone issue bracketing out any broader concerns. The second is interested in reconciling the goals of healthcare with mitigation burdens. When considering healthcare and climate change mitigation we can adopt either:

1. Isolationism: Separate and treat each goal in isolation. One way to isolate these goals is to formulate and apply principles that surround each goal separately. One principle would determine mitigation responsibilities without consideration of healthcare’s role in social justice, like a polluter pays principle. Alternatively, principles of justice can be applied to healthcare in isolation of environmental considerations, i.e. maintain the status quo.
2. Integrationism: formulate principles of justice that help balance and integrate the goals of healthcare and mitigation responsibilities.

Based on the distinction between isolationism and integrationism we can place these issues on a spectrum. This spectrum essentially tracks the degree to which healthcare emissions are treated differently. At one end, we insulate the goals of healthcare from mitigation burdens and treat the goals of healthcare as an exemption from mitigation burdens. That is, we say healthcare is special and healthcare emissions are different because they are essential for providing the benefits of healthcare. I call this view ‘absolute healthcare exceptionalism’. At the other end, lies a different isolationist position where we allocate mitigation burdens independently of the goals of healthcare. Healthcare is regarded as no different to any other polluter and the purpose of healthcare emissions are irrelevant to how mitigation burdens are allocated. I call this view ‘healthcare non-exceptionalism’. Between these isolationist views lies a degree to which exceptions are made for healthcare depending on how these ideals are conjoined. In theory, there are several ways of integrating these goals which I discuss later in Section “Methods of isolation”. I argue for one moderate position which attempts to balance these two potentially competing issues.

## Methods of isolation

Over the course of this section I discuss each isolationist stance. Two examples of isolationism are considered. As I mentioned above, I contrast ‘healthcare non-exceptionalism’, the standpoint that mitigation burdens should be allocated to healthcare independently of its goals, with ‘absolute healthcare exceptionalism’, the position that healthcare should be exempt from mitigation because healthcare is special. Although isolationism is the shared underlying methodology, these views come down quite differently on the extent that healthcare emissions should be treated differently. For each argument I provide separate reasons to reject these. What is clear from these arguments however are the important connections between health, healthcare and climate change. As these issues of distributive justice are interconnected, it is very difficult to separate out how we think about healthcare’s emissions, what healthcare should do regarding climate change and how to provide the benefits of healthcare.

One might be tempted to think of these issues in Walzerian terms as separate spheres of justice (Walzer 1983). Famously, Walzer defends the idea that a shared understanding of goods like health, education, wealth, political power and so forth determine how they are distributed. The result is that different goods are distributed using different principles. Each sphere has a corresponding principle of distribution, health is distributed based on need, wealth by the market and so forth. A central concern of Walzer’s is that each sphere is prevented from dominating another. The meaning and understanding of one social good isn’t used to shape the intrinsic meaning of another (Walzer 1983, 10–11). So wealth, which is distributed by market ideals, shouldn’t be used to buy health since the social meaning of health dictates this should be distributed by need. Thus, isolationist views on healthcare exceptionalism could be a way of preventing issues of climate change dominating the distribution of health or vice versa.

It is not obvious that there is a shared understanding over climatic responsibilities with a resulting sphere of justice (Caney 2018). More importantly, even if healthcare should be distributed by need as Walzer suggests, emissions are required to meet healthcare needs. Healthcare emissions reflect how healthcare meets its goals and what principles of justice are adopted for distributing healthcare resources. As the sphere of health has implications for emissions through healthcare it is therefore difficult to see how these issues can in principle be kept separately. In a similar vein, emissions have consequences for health, and healthcare systems will increasingly have to respond to the health threats posed by climate change. Again, on a Walzerian view it is hard to see how these issues can be isolated into separate spheres.

I raise the example of separate spheres to provide an overview of how isolationism might work and why it should be rejected. However, it is important to consider these arguments in more detail and so I discuss both healthcare non-exceptionalism and absolute healthcare exceptionalism next.

### Healthcare non-exceptionalism

Let us turn to healthcare non-exceptionalism, the view that mitigation responsibilities should be allocated based on criteria that make no reference to the goals of healthcare.

One key principle for sharing mitigation burdens is a polluter pays principle (PPP). The PPP is widely discussed when it comes to allocating mitigation burdens, and is frequently endorsed by economists (Caney 2006; Cripps 2013; Meyer and Roser 2010; Page 2008; Shue 2014; Vanderheiden 2008). This principle is an intuitive way of allocating responsibilities to address climate change and is familiar from other moral and legal practices as we generally consider it to be fair when the one causing a problem is the one who fixes it. The PPP is a principle of causal responsibility and assigns responsibility based on, and to the extent that, one is a polluter (Shue 2014, 182–183). As a contribution-based principle, the PPP is a principle of formal, as opposed to substantive, equality. Principles of formal equality have two components: equality as universality where a principle applies to all in the same way, and equality as impartiality meaning that we treat like cases alike (Gosepath 2021). All polluters are identified and treated the same by the PPP. If one is producing emissions the PPP is exclusively interested in recognising one as a polluter and quantifying their emissions such that remedial responsibilities can be allocated in proportion to pollution.

Under a PPP, the goals of healthcare and the purpose of healthcare emissions are irrelevant to whether, and to the extent that, healthcare should undertake mitigation. Equality as impartiality leads the PPP to treat like cases alike, where the criterion of interest is being a polluter and likeness is determined emissions. The PPP is not interested in any other factors and so as a polluter healthcare is met with neither favour nor discrimination. The PPP is therefore insensitive to the goals of healthcare. All that matters for a PPP is that healthcare is in fact a polluter, meaning that the PPP is isolationist. Furthermore, because the goals of healthcare provide no reason to treat healthcare as exceptional on a PPP, I adopt the label ‘healthcare non-exceptionalism’.

The problem with setting any wider considerations of justice aside via a PPP are that two forms of injustice result: that mitigation costs are disproportionate and unfairly distributed. Demanding healthcare systems pay in proportion to emissions without consideration of the purpose of those emissions is overly demanding. If healthcare systems are responsible for all their emissions, the result is that both historic as well as current emissions must be accounted for. The NHS, by way of illustration, was established in 1948. Whilst there is no empirical data for NHS emissions stretching back this far, nor modelling of exactly what this would cost for the NHS, it seems reasonable to assume that mitigation costs would be substantial. Emissions dating between 1990 and 2019 equate to approximately 1 gigaton of CO_2_ equivalent for the NHS in England (Tennison et al. 2021). Mitigation costs in proportion to emissions from healthcare that we can measure are likely to be extensive never mind those stretching back further.

Indeed, the above may partly explain why many healthcare systems, including the NHS, have committed to a target of net-zero rather than a strict target accounting for all greenhouse gas emissions (National Health Service 2020; World Health Organisation 2024). Net-zero requires emissions neutrality: any emissions must be counterbalanced by offsets. Net-zero is also forward-looking, aiming to bring current emissions down and then offset the remainder which offers much more flexibility in how mitigation is achieved. The PPP, however, is backwards-looking and healthcare is liable for all its historic as well as current emissions. Whether net-zero is the policy for polluters is open to debate (Welton 2022), the critical point here is that since mitigation is burdensome and there is a concern about how healthcare meets its primary goals whilst reducing emissions, it is better to aim for emissions neutrality which requires substantially less than mitigating all one’s emissions.

One may object that addressing climate change isn’t in principle different to any other large cost for healthcare systems.^3^ For instance, there are legal requirements that carry burdens for healthcare systems but are part of meeting individual’s fundamental entitlements like pay for parental leave. We don’t, however, make an exception for healthcare even if such requirements are very burdensome. Something similar might be said for GHG mitigation. It may be burdensome for healthcare, but if that’s what it takes to protect people from the threats of global warming then it is simply another cost for healthcare.

This is a powerful objection to making climate change mitigation a special case where healthcare should be treated differently to other polluters. However, even if we do not think that the magnitude of mitigation burdens for healthcare should effect their fundamental responsibilities to address their emissions, we may be concerned that healthcare non-exceptionalism results in a *distribution* of mitigation burdens that is unfair.

The second problem then concerns not the size of mitigation burdens but their distribution. It would be unfair if mitigation costs were to fall disproportionately on those who are disadvantaged, or who in general terms contribute less to climate change. Such a situation is a potential result of adopting a PPP in healthcare. Bhopal and colleagues plotted healthcare’s carbon footprint as a proportion of total per capita carbon footprint by decile (Bhopal et al. 2022). They found that emissions follow a social gradient where the poorest decile in England use 20% of their carbon emissions on healthcare whereas the wealthiest decile spend 10 times less (2%) of their total carbon emissions this way. In general, wealth is strongly associated with greater emissions (Chancel 2022). According to Bhopal and colleagues the wealthiest 10% in England emit around 28 tonnes of CO_2_ equivalent annually compared to 3 tonnes from the poorest. In global terms, the bottom 50% of the world’s population were responsible for 12% of global emissions as opposed to 48% of emissions coming from the wealthiest 10% in 2019 (Chancel 2022).

What Bhopal and his co-authors demonstrate is how those who in general emit the least, and who are already most disadvantaged, are most vulnerable to policies to reduce the carbon footprint of healthcare because a greater proportion of their emissions are wrapped up in healthcare. Since the PPP allocates mitigations burdens in proportion to emissions, the greatest emitters should do the most. And as those who pollute the most tend to be wealthy, then a PPP would usually shift burdens on to the most advantaged. However, this situation becomes flipped when applied to healthcare. Emissions in healthcare follow the greatest need, and those with the greatest need tend to be disadvantaged. But when the PPP brackets out the purpose of healthcare, or any wider concerns of justice, and simply says ‘polluters should pay in proportion to their emissions’, in healthcare this results in the costs falling disproportionately on those who are disadvantaged and who contribute the fewest emissions overall.

Worse still, economic inequality is associated with worse health outcomes (Marmot et al. 2020), and even in the UK it is the disadvantaged who are most vulnerable to the health effects of climate change (Paavola 2017). Consequently, asking healthcare to decarbonise in proportion to their emissions risks asking those who are poor, suffer ill health, contribute the least to global warming and stand to lose the most from its effects to make the greatest sacrifices. This is unfair. At the very least, healthcare decarbonisation efforts ought to be sensitive to pre-existing inequalities as well as the distribution of the benefits of healthcare and the burdens of mitigation. Healthcare non-exceptionalism is incapable of this because it is concerned exclusively with mitigation.

### Absolute healthcare exceptionalism

To guard against the injustices of a PPP we could look to the other face of isolationism, namely ‘absolute healthcare exceptionalism’. One possibility is to rely on the idea that healthcare is special in order to treat healthcare differently in terms of any wider concerns of justice, including mitigation burdens.

Achieving climatic targets do not necessarily mean that all must contribute equally. If some mitigate to a greater extent or at a faster rate, it is still possible, though increasingly difficult, to keep global warming below 1.5–2 °C while others emit. As a target of 1.5 °C is compatible with a certain budget of greenhouse gas emissions (Rogelj et al. 2016), that budget could be distributed such that some actors emit as long as others pull in the slack. Similarly, net-zero is possible at a national or global level with some emitting so long as the emissions books are balanced by the mitigation efforts of others.

One reason to exempt healthcare from mitigation responsibilities is because it is special. Philosophers have tended to discuss the idea that healthcare is special with regards to wealth inequality. For example, Segall describes the specialness thesis as: “to say that healthcare is special is to say that it is morally significant in ways that justify distributing medical resources *in isolation* from the way in which other social goods, and wealth in particular, are distributed [my emphasis]” (Segall 2007, p. 343). Fundamentally, the specialness thesis is about treating healthcare differently when it comes to issues of distributive justice. The basic idea is that how healthcare resources are used to organise, structure and deliver healthcare should be done separately from considerations of the just distributions of other social goods (and bads). Indeed, this is typical of how healthcare resources are allocated currently; they are isolated from wider concerns of justice in so far as they are concerned with particular health distributions amongst narrowly defined populations over relatively short time horizons (Albertsen and Knight 2015; Munthe et al. 2021; Peter 2001). For instance, a health system in Greater Manchester is concerned with, say, maximising health for those living in Greater Manchester or reducing health inequalities amongst residents of Greater Manchester. Climate change has a far wider international and intergenerational perspective, however. As healthcare is special, we can treat it as exceptional when it comes to the distribution mitigation burdens and benefits.^4^

Daniels offers the most influential argument for the specialness thesis (Daniels 2007). Daniels claims healthcare is special because of its role in protecting and promoting health. Health, read as species typical normal functioning (Boorse 1977), holds strategic importance for protecting one’s share of the ‘normal opportunity range’ according to Daniels. Borrowing from a Rawlsian conception of justice as fairness, Daniels makes the case for an egalitarian distribution of opportunities. As health protects opportunity and healthcare protects health, healthcare, according to Daniels, is afforded special moral importance as per the specialness thesis (Daniels 2007, p. 49).

It became apparent to Daniels, however, that the social determinants of health like working conditions and income inequality had a far greater impact on health than healthcare. Healthcare was, so to speak, “the ambulance waiting at the bottom of the cliff” (Daniels 2007, p. 79). In response, Daniels adjusted his theory maintaining the central position of health in protecting the normal opportunity range, but Daniels extended the scope of the specialness thesis to cover any health need. Both those heath needs customarily dealt with by healthcare as well as those identified by the social determinants of health were included. So, the specialness thesis can be revised: to say that health is special is to say that it is morally important in ways that justify distributing resources that meet health needs like healthcare and the social determinants of health in isolation from other concerns of distributive justice.

Daniels does not make the case that environmental determinants of health are also a health need and fall under the specialness thesis. However, it is a plausible extension of his arguments and would mean environmental determinants of health, like the social determinants, are special.

Climate change is predicted to have significant impacts on health, mediated through environmental determinants of health, as well as compounding social determinants like increasing poverty for instance (Haines and Ebi 2019; Haines and Patz 2004). The World Health Organisation estimates that between 2030 and 2050 climate change will lead to approximately 250 000 additional deaths per year from malaria, diarrhoea and heat stress (World Health Organisation 2023). Not to mention the effects extreme weather has for air quality, crop survival, drinking water and habitable areas (Watts et al. 2015). Some claim that “climate change is the greatest threat to public health in the 21st century” (Costello et al. 2009). As climate change threatens health, then tackling climate also meets a health need. As a health need, climate change mitigation would therefore also fall under the specialness thesis.

This is where arguments like Daniels, that want to treat healthcare as special because of its role in protecting and promoting health, run into trouble. Absolute healthcare exceptionalism rests on treating healthcare as special because it protects health needs and therefore is exempt from mitigation burdens. Mitigation efforts, by reducing climate change threats, also address health needs, specifically the environmental determinants of health. If health needs are special, including those met by healthcare, the social determinants of health and the environmental determinants of health, then mitigation is also special. So, the specialness thesis could be taken to imply that healthcare is special and exempt from mitigation burdens. But, by the same token, the specialness thesis also suggests that mitigation is special because it meets health needs, challenging the idea that healthcare should be exempt from mitigation. The specialness thesis implies both that healthcare is treated differently when it comes to mitigation and that it is not.

Once we acknowledge that healthcare generates emissions, and these emissions can contribute to health needs, we cannot allocate mitigation burdens separately to healthcare on the basis that health, and therefore healthcare, is special. Given that the social determinants of health make it difficult to construct a theory of health justice in isolation from general considerations of justice (Segall 2007, 2010; Wilson 2009), it becomes even harder to do this when it comes to the environmental determinants of health given the complex relationship between health needs, healthcare, emissions from healthcare and climate change. Any view that seeks to exempt healthcare from mitigation on the basis that healthcare protects health will face the difficulty of justifying this when mitigation also contributes indirectly to health. Indeed, if climate change is the greatest threat to public health this century, it may well be that climate change mitigation, including for healthcare systems, does more to protect health than healthcare alone.

## Methods of integration: moderate healthcare exceptionalism

Allow me a brief recap. I am concerned with the relationship between two issues in distributive justice: the goals of healthcare and the allocation of mitigation responsibilities. Their relationship raises a question of whether, and to what extent, we should make an exception to mitigation responsibilities for healthcare. I have claimed that there are two ways to understand this relationship. We can separate these distributive concerns or attempt to integrate them. The isolationist method leads us to argue either for absolute healthcare exceptionalism where there is an exemption for healthcare, or healthcare non-exceptionalism where mitigation responsibilities are allocated on criteria independent of healthcare’s role. I have rejected these. This leaves integrationism. If, when assessing what a fair share of the burdens of tackling climate change are for healthcare systems, we cannot ignore the morally valuable role of healthcare but nor can we exempt healthcare, then they must be balanced. This is the next task of this paper.

Before going on to make my argument for when it would be justified to make exceptions to mitigation burdens for healthcare, I want to make a few comments on integrationism. Caney makes a further distinction between moderate and strong integrationism (Caney 2018). The main difference between these is the scope of goods that each considers. “Moderate Integrationism: This holds that we should apply principles of justice to a good X, but in doing so we should also take into account other considerations.” Whereas strong integrationism applies a general principle of justice to a whole package of benefits and burdens that include a good X.^5^

Now, as my concern is with thinking about how to combine two issues of distributive justice, healthcare and climate change, I am operating under the auspices of moderate integrationism.^6^ However, Caney argues in favour of strong integrationism because of the way that climate change is wrapped up in a whole host of distributive concerns (Caney 2012, 2018). The issue for my purposes is that, as compelling as a comprehensive theory of individuals’ just entitlements that account for the global and intergenerational nature of climate change might be, it is lacking practical force. For healthcare systems faced with the question of how to decarbonise fairly, pointing to, say, a global difference principle does not provide much practical guidance in how to reconcile the competing concerns of providing quality care whilst minimising emissions.

One further, and final, note on method. As the approach taken here is a moderately integrationist one, there are two ways that we could view one distributive issue as our primary concern whilst also factoring in a second consideration. One way is to start with justice in health and factor in concerns about climate change. Alternatively, we could run this the other way, starting with an account of mitigation responsibilities and making concessions for healthcare. In the first, bottom-up method, given that both healthcare and climate change mitigation contribute to health, we could start with the question of why health matters to justice and work towards the emissions that are compatible with meeting individuals’ just entitlements to health. In theory, a bottom-up method could be used to derive a healthcare system’s permissible emission. With the space I have remaining, I want to say something relatively practical and so my argument takes a top-down approach. That is, I start from mitigation responsibilities and work in justified exceptions to this on the basis that healthcare has a valuable function.

### Ability to pay

An ability to pay principle (APP) can accommodate some mitigation exceptions for healthcare without necessarily providing an exemption. An APP is often used to allocate mitigation burdens fairly (Shue 2014, 186–189). The idea being that those with the greatest capacity to shoulder the burdens of climate change mitigation should. According to Miller, the APP is a principle of *capacity* where “remedial responsibilities ought to be assigned according to the capacity of each agent to discharge them.” (Miller 2001) Capacity is usually interpreted in the climate context as wealth (Page 2008). Remedial responsibilities are those responsibilities that we have to remedy some injustice.

Miller starts from the thought that, amongst a pool of potential duty-bearers, an APP can be used to determine who is best placed to act (Miller 2001). Miller imagines a rescue case. Several potential candidates could save an endangered swimmer, the question is how to figure out who? Determining who is best placed to undertake the rescue is based on two criteria: capacity as effectiveness and capacity as cost. Capacity as effectiveness leads to ranking swimmers according to swimming strength. Capacity as cost is then used to sort through candidates where the strongest swimmer might be ruled out if the costs are too great. Accordingly, responsibility then falls to the next most able. Who is best placed becomes a ratio of most effective with the least costs.

Miller is correct in identifying cost and effectiveness as the relevant criteria in the APP. However, his concern is to single out *the* agent who is best placed to perform some remedial action. Here, I use an APP slightly differently. Rather than seeing the APP in more of a binary way as Miller does, where individuals either do or not have an ability to pay, I take it to be a scalar concept. Instead of asking who is responsible to undertake remedial actions X, my view asks, ‘what can agents do to help towards X?’. What agents can do is shaped by their effectiveness and the costs to them in contributing to solving some problem.

Tackling climate change is a collective issue. Adequate action to mitigate the threats of climate change will require a response from many actors and institutions. My default assumption is that all, including healthcare, have some responsibility to undertake mitigation. There are potentially three reasons we could assume healthcare has a *prima facie* responsibility to mitigate. First, as mentioned in section “Absolute healthcare exceptionalism”, climate change has impacts on health. The second reason could refer to healthcare’s emissions as a reason to say healthcare ought to do something without yet specifying precisely what that something is, unlike a PPP. The third is effectiveness. Climate change mitigation is most effective if we start from a default that all must mitigate unless we have reasons to rule them out.

The question then is not whether healthcare should mitigate or not, but what can healthcare do to tackle climate change? This is where an APP comes in to help provide both the degree to which, and the limits upon, mitigation responsibilities. Like the PPP, the APP also relies on one aspect of formal principles of equality: equality as universality. All, *prima facie*, have some ability to mitigate. Where the APP diverges from a PPP however is in equality as impartiality. On casual principles, like a PPP, if A caused a harm to B, then A should pay regardless of costs (Miller 2001). The APP is partial, however. What agents do increases in line with effectiveness and their ability to bear the costs. Those who are less effective, or where action is too costly, do less. When an agent’s action would be more effective and less costly all things considered, they are expected to do more. The appeal of the APP is the way that, in asking not just what would be effective when trying to bring about a goal, but who is able to bear the costs of doing so, it can be adjusted for the position or valuable social role of potential contributors.

Allow me to specify what I mean by effectiveness and costs regarding policies to decarbonise healthcare. Effectiveness depends, in part, on the goals on adopts. If effectiveness is measured in general ways like ‘to prevent climate change’, the goal is unrealistic. For climate mitigation, the goal is simply to reduce one’s emissions. Costs for a healthcare system are in reference to its capacity to meet its primary goal of protecting and promoting health, the relief of suffering, prolonging life and the like. Whilst the APP is typically interpreted in terms of wealth when it comes to climate change - the wealthy should pay most - health being the primary function of healthcare makes this a more appropriate way to consider reasonable costs.

To get more precise on what the mitigation responsibilities of healthcare systems are, I specify the point at which healthcare systems have an *in*ability to pay. That is, the limit at which healthcare systems are excused from mitigation burdens. It is one thing to suggest that healthcare systems can take various actions to limit emissions, with adjustments based on effectiveness and cost. However, to avoid vagueness about what this entails in practice and to better integrate mitigation efforts with healthcare goals, it is necessary to specify a threshold beyond which emissions are no longer justified. In other words, it is important to say when healthcare cannot mitigate, as well as suggesting when it can. Beyond this limit, the costs for healthcare systems are disproportionate at which point we can say that healthcare has an *in*ability to pay and thus is not expected to shoulder mitigation burdens. Although it could be the case that ineffectiveness also provides a reason to say that a healthcare system has an inability to mitigate, this circumstance is unlikely in practice. Ineffectiveness suggests that an act is not likely to reduce emissions. But we can always reduce emissions by ceasing to perform the emitting act, the reason we do not is because of the costs in failing to realise an important goal. Hence, capacity as cost is the predominant threshold in determining an inability to pay.

### Specifying the inability threshold

Many theorists accept that there is some limit on what costs we should accept when it comes to averting climate-mediated harms (Duus-Otterström 2023; Rao and Baer 2012; Shue 1993; Vanderheiden 2008, p. 243). One way to demarcate that limit is through a distinction between luxury and substance emissions. As emissions themselves are of instrumental importance, Shue points out the importance of distinguishing “the fact that some sources [of greenhouse gas emissions] are essential and even urgent for the fulfilment of vital needs and other sources are inessential or even frivolous.” (Shue 1993) For Shue those emissions that are necessary to protect a basic need are “subsistence” whereas everything else he calls “luxury”. For healthcare, a dichotomy of luxury emissions on the one hand and subsistence on the other is a little coarse. Nevertheless, the concept of subsistence emissions serves as a useful threshold on those healthcare emissions that we make an exception for whilst accepting that all other emissions are treated differently and liable to mitigation costs.

The limit on mitigation burdens for a healthcare system should lie where emissions are necessary to protect something of fundamental moral value. Subsistence emissions have two necessary and jointly sufficient features: (i) emissions must satisfy a basic need; and, (ii) the emissions must be necessary to achieve that (i.e. there must be no reasonable way of achieving the same end with fewer emissions) (Duus-Otterström 2023). Basic needs are often taken to be a subset of humans’ most fundamental needs without which they would be harmed (Wiggins 1987). Some level of health is, on most accounts, of moral value because of the role it plays in securing opportunity, well-being or flourishing for example (Daniels 2007; Nordenfelt 2006; Powers and Faden 2008; Venkatapuram 2011). As such, at least some activities of healthcare would widely be considered to meet a basic need. However, to be considered subsistence, the greenhouse gases emitted in securing basic healthcare needs must also be the minimum necessary.

An example serves to highlight the difference between subsistence and luxury emissions. Consider metered-dose inhalers (Parker 2022). These inhalers are used to treat respiratory illness but contain powerful greenhouse gases (Janson et al. 2020; Wilkinson and Woodcock 2022). Most would agree that managing respiratory problems like asthma is a valuable goal and would help secure individual’s basic needs. So metered-dose inhalers pass the first test: they meet a basic need. The follow up is whether these emissions are *necessary* to achieve the end of treating respiratory disease. The question as to whether they are necessary, however, depends on the characteristics of the patient requiring treatment. Some patients can use alternative inhalers which do not contain greenhouse gases. For those who cannot use an alternative, the greenhouse gases emitted when they use a metered-dose inhaler are subsistence emissions and therefore permissible. There is no alternative way of meeting the same end of protecting their respiratory health with fewer emissions. Switching inhalers amongst those who can then ensures that emissions are the minimum necessary.

Clearly the inhalers example is highly simplified and is itself somewhat exceptional in terms of healthcare mitigation because higher carbon inhalers can often be straightforwardly switched. In some instances this may actually be *better* for patient care, and oftentimes it is no worse, though this is not to say switching is always without burdens (Parker 2022). As much as inhalers provide a useful example of how policy could be drawn from the APP, we might worry that in more challenging, and more typical cases of healthcare decarbonisation, the APP is insufficiently action-guiding.^7^ In section. “Preliminaries” I alluded to the fact that there is an extensive and diverse range of actions that healthcare systems can undertake to reduce their emissions which requires reconfiguring services, investments in lower carbon technologies, focusing on disease prevention rather than treatment and so forth. It may be that in pursuing these actions there are not always substitutions that leave health unaffected and basic needs met, as is regularly the case with inhalers.

The APP does not necessarily rule out any of these actions and indeed the variety of things healthcare can do to reduce its carbon footprint highlights that there is a huge opportunity for healthcare systems to radically reduce their carbon footprint. Nevertheless, the APP places a threshold on what level of burdens should be accepted by agents, and in particular here how much health they should be required to sacrifice, in order that healthcare systems mitigate their emissions. It is difficult to review every instance of a mitigation policy to assess whether the costs are excessive in a paper of this nature. But the APP provides an overarching principle to guide the extent of healthcare’s responsibilities and how healthcare systems can reconcile protecting and promoting health with sustainability. Three questions can be drawn from the APP to help guide healthcare mitigation policy.

Take mitigation policies like replacing ambulances with electric versions or installing photovoltaics. The first question is whether they meet a basic need? Clearly ambulances are required to protect health, and healthcare systems have energy requirements that could be met, in part, through photovoltaics. Indirectly at least, ambulances and photovoltaics are part of meeting basic needs through healthcare. Where healthcare systems are producing emissions that are not directed to meeting basic needs there are strong reasons to address these emissions. The next question is whether they are the minimum reasonably necessary. Again, an electric ambulance appears to generate the minimum necessary emissions that are reasonable in attending emergencies and transporting patients. Of course, bicycles and sending paramedics on foot would produce fewer emissions, but this would be an unreasonable way of meeting the needs identified. The final question is whether replacing the ambulance fleet with electric vehicles or changing energy infrastructure presents an unreasonable or excessive cost. This is possibly the most complex issue requiring greater empirical data than is currently available. However, the test we should apply is whether meeting these costs would prevent healthcare systems securing sufficient health. At this point, we can say that healthcare has an inability to pay.

In sum, an APP in terms of healthcare is more concerned with when healthcare systems have an *in*ability to pay. Phrased in the negative we start from the observation that there are various ways for healthcare systems to curb their emissions, but that we say the costs are excessive where it asks healthcare to further mitigate emissions that are already the minimum necessary to meet certain valuable goals. In this way, we can combine mitigation responsibilities with the morally valuable goals of healthcare demarcating the limits on which healthcare systems should and should not mitigate given these dual, and potentially conflicting, goals.

## Conclusion

Theories of social justice frequently concern themselves with the fair distribution of health. Recently, attention has shifted from healthcare alone to consider how other social bases of health contribute to health justice. The challenge now is to examine how healthcare not only contributes to health but how complex and technologically advanced healthcare systems simultaneously undermine health through climate change. I have argued that the key to understanding healthcare’s role in mitigation is an exploration of the ways that healthcare, and mitigation burdens, are exceptional.

Various ways of viewing mitigation burdens and healthcare systems as exceptional are possible. Here I explored several potential views based on a distinction between isolationism and integrationism. I have argued against isolationist approaches that treat these goals as separate. One conclusion from my analysis is that theories of health justice must accommodate climate change mitigation. I have provided one such way of doing this by taking a moderate integrationist stance that relies on an ability to pay principle. An ability to pay principle provides the degree that healthcare should engage in mitigation by highlighting the limits to this responsibility. This allows policies that address climate change to be sensitive to the value of the role of healthcare without making healthcare exempt.

The strength of my view lies in it being relatively practical by offering guidance on how to balance the potentially conflicting demands of both reducing healthcare emissions whilst still providing quality care. This is important because how policy makers, hospital managers and health professionals determine when and the ways that healthcare or climate change burdens are exceptional will shape the kinds of healthcare systems that societies have. Nevertheless, it may be that pragmatic solutions do not align well with a comprehensive theory of just distributions. One important implication of my arguments regarding exceptionalism is how healthcare climate policies sit with ideals of a just distribution and how to reconcile these issues of distributive justice with a need for healthcare systems to take robust action on their emissions.

## Footnotes

1 That is not to say that these two goals can never coincide. Examples include waste reduction or avoiding activities like “overdiagnosis” that are not thought to contribute to the goals of healthcare. Where reducing the emissions of healthcare makes no difference to the distribution of benefits and burdens provided by a healthcare system, there is no question of justice to answer. However, these goals may not always coincide and there is still a question of transitional justice in terms of how healthcare systems shift away from these activities. Hence, I assume that there are areas where reducing healthcare emissions are in tension with providing the benefits of healthcare.

2 As an anonymous peer reviewer points out, people may also have *unreasonable* concerns about healthcare reducing its carbon footprint. For example, because they believe that climate change is a hoax. Those who believe climate change is a hoax, or who depart significantly from the scientific consensus on climate change are unlikely to be moved by my arguments here. Their objections to healthcare, or any other institution or individual for that matter, reducing their greenhouse gas emissions would have to be dealt with quite differently to the approach I take here.

3 I am grateful to an anonymous peer reviewer for pushing me on this point.

4 As a minor point of clarification, there are two further considerations. One is to say that that neither Segall, nor others who endorse the specialness thesis like Norman Daniels, view health as the most important good. For Daniels, opportunity is the most important good, not health or anything else. (Daniels 2007, p. 57) Furthermore, the separateness thesis treats healthcare as a separate sphere of justice. (Walzer 1983) This separateness thesis builds on the idea of specialness to argue that only medical criteria should be used in allocating healthcare resources. (Brock 2003) Charting a path from the separateness thesis to absolute healthcare exceptionalism is straightforward enough. If healthcare is a separate sphere and forbids allocation on the basis of non-medical criteria like wealth, then as mitigation burdens are non-medical criteria, they are simply irrelevant and thus healthcare is exempt. The separateness thesis is, however, more demanding as it entails the specialness thesis. As the specialness thesis does not rely on the separateness thesis, and the separateness thesis is likely to be less widely acceptable as it is more demanding, I focus on the specialness thesis. (Lippert-Rasmussen and Lauridsen 2010; Persad and du Toit 2020) However, much of what I have to say is relevant to the separateness thesis.

5 In particular, Caney writes, “Strong Integrationism: This holds that we should treat X merely as one element in the total package of burdens and benefits and then this total package should be regulated by a general principle of justice (such as a global difference principle or a commitment to basic rights).”(Caney 2018).

6 There is a further issue regarding integrationism. To what extent should various issues of justice should in healthcare be addressed together? Healthcare systems are facing a raft of challenges that raise issues of distributive justice and how we structure and organise healthcare systems beyond just climate change mitigation. Post-COVID recovery, meeting rising demands for healthcare services, aging populations, the ever-increasing cost of new technologies and treatments, stalling life-expectancies in high-income nations and so forth. As healthcare systems change and adapt to these challenges, there is an issue of the extent to which these should be integrated with how healthcare systems address climate change. I simply note these here and limit myself to integrating healthcare mitigation with the goals of healthcare.

7 An anonymous peer reviewer helpfully raises this point.

## References

[CR1] Albertsen, Andreas, and Carl Knight. 2015. A Framework for Luck Egalitarianism in Health and Healthcare. *Journal of Medical Ethics* 41(2): 165–169. 24505116 10.1136/medethics-2013-101666

[CR2] Bhopal, Anand, Kristine Bærøe, and Ole F. Norheim. 2022. How do we decarbonise fairly? Emissions, inequities and the implications for net zero healthcare. *Journal of the Royal Society of Medicine* 115(9): 337–340. 10.1177/01410768221113069 10.1177/01410768221113069 PMC9634230 35944580

[CR3] Boorse, Christopher. 1977. Health as a theoretical concept. *Philosophy of Science* 44(4): 542–573.

[CR4] Brock, Dan W. 2003. Separate spheres and Indirect benefits. *Cost Effectiveness and Resource Allocation: C/E* 1(1): 4. 10.1186/1478-7547-1-4 12773217 10.1186/1478-7547-1-4 PMC156023

[CR5] Brülde, Bengt. 2001. The goals of medicine. Towards a unified theory. *Health Care Analysis* 9(1): 1–13. 10.1023/A:1011385310274 11372571

[CR6] Caney, Simon. 2006. Cosmopolitan justice, rights, and global climate change. *Canadian Journal of Law and Jurisprudence* 19(2).

[CR7] Caney, Simon. 2012. Just emissions. *Philosophy and Public Affairs* 40(4): 255–300.

[CR8] Caney, Simon. 2018. Distributive Justice and Climate Change. In *Oxford Handbook of Distributive Justice*, ed. Serena Olsaretti. Oxford: Oxford University Press. 10.1093/oxfordhb/9780199645121.013.23

[CR9] Chancel, Lucas. 2022. Global Carbon Inequality over 1990–2019. *Nature Sustainability* 5(11): 931–938. 10.1038/s41893-022-00955-z

[CR10] Costello, Anthony, Mustafa Abbas, Adriana Allen, Sarah Ball, Sarah Bell, Richard Bellamy, Sharon Friel, Nora Groce, Anne Johnson, and Maria Kett et al. 2009. Managing the health effects of climate change: lancet and University College London Institute for Global Health Commission. *Lancet (London, England)* 373(9676): 1693–1733. 10.1016/S0140-6736(09)60935-1 10.1016/S0140-6736(09)60935-1 19447250

[CR11] Cripps, Elizabeth. 2013. *Climate change and the moral agent: Individual duties in an interdependent world*. Oxford University Press.

[CR12] Daniels, Norman. 2007. *Just health: Meeting health needs fairly*. Cambridge University Press.

[CR13] Duus-Otterström, Göran. 2023. Subsistence emissions and climate justice. *British Journal of Political Science* 53(3): 919–933. 10.1017/S0007123422000485

[CR14] Eckelman, Matthew J., and Jodi Sherman. 2016. Environmental impacts of the U.S. health care system and effects on public health. *PLOS ONE* 11(6): e0157014. 10.1371/journal.pone.0157014 10.1371/journal.pone.0157014 PMC4900601 27280706

[CR15] Gosepath, Stefan. 2021. Equality (Stanford Encyclopedia of Philosophy). https://plato.stanford.edu/entries/equality/

[CR16] Haines, Andy, and Kristie Ebi. 2019. The imperative for climate action to protect health. *New England Journal of Medicine* 380(3): 263–273. 10.1056/NEJMra1807873 30650330 10.1056/NEJMra1807873

[CR17] Haines, Andy, and Jonathan A. Patz. 2004. Health effects of Climate Change. *Journal of the American Medical Association* 291(1): 99–103. 10.1001/jama.291.1.99 14709582 10.1001/jama.291.1.99

[CR18] Hastings Centre. 1996. The goals of medicine. *Hastings Center Report* 26(6): S1–S27. 10.1002/j.1552-146X.1996.tb04777.x 8970793

[CR19] Health Care Without Harm. 2019. Health care’s climate footprint: How the health sector contributes to the global climate crisis and opportunities for action. https://noharm-global.org/sites/default/files/documents-files/5961/HealthCaresClimateFootprint_092319.pdf

[CR20] Janson, Christer, Richard Henderson, Magnus Löfdahl, Martin Hedberg, Raj Sharma, and Alexander J. K. Wilkinson. 2020. Carbon footprint impact of the choice of inhalers for asthma and COPD. *Thorax* 75(1): 82–84. 10.1136/thoraxjnl-2019-213744 31699805 10.1136/thoraxjnl-2019-213744 PMC6929707

[CR21] Lenzen, Manfred, Arunima Malik, Mengyu Li, Jacob Fry, Helga Weisz, Peter-Paul Pichler, Leonardo Suveges Moreira Chaves, Anthony Capon, and David Pencheon. 2020. The environmental footprint of health care: A global assessment. *The Lancet Planetary Health* 4(7): e271–e279. 10.1016/S2542-5196(20)30121-2 10.1016/S2542-5196(20)30121-2 32681898

[CR22] Lippert-Rasmussen, Kasper, and Sigurd Lauridsen. 2010. Justice and the allocation of healthcare resources: Should indirect, non-health effects count? *Medicine, Health Care and Philosophy* 13(3): 237–246. 10.1007/s11019-010-9240-9 20306307

[CR23] Marmot, Michael, Jessica Allen, Tammy Boyce, Peter Goldblatt, and Joana Morrison. 2020. *Health equity in England: The marmot review 10 years on*. Institute of Health Equity. https://www.health.org.uk/sites/default/files/upload/publications/2020/Health%20Equity%20in%20England_The%20Marmot%20Review%2010%20Years%20On_full%20report.pdf

[CR24] Meyer, Lukas H., and Dominic Roser. 2010. Climate justice and historical emissions. *Critical Review of International Social and Political Philosophy* 13 (1): 229–253.

[CR25] Miller, David. 2001. Distributing responsibilities. *Journal of Political Philosophy* 9 (4): 453–471.

[CR26] Munthe, Christian, Davide Fumagalli, and Erik Malmqvist. 2021. Sustainability principle for the ethics of healthcare resource allocation. *Journal of Medical Ethics* 47(2): 90–97. 10.1136/medethics-2020-106644 10.1136/medethics-2020-106644 PMC7848061 33154090

[CR27] National Health Service. 2020. Delivering a ‘Net Zero’ National Health Service. https://www.england.nhs.uk/greenernhs/publication/delivering-a-net-zero-national-health-service/

[CR28] Naylor, Chris, and John Appleby. 2012. Sustainable health and social care: Connecting environmental and financial performance. https://www.kingsfund.org.uk/publications/sustainable-health-and-social-care

[CR29] Nordenfelt, Lennart. 2006. The concepts of health and illness revisited. *Medicine, Health Care and Philosophy* 10(1): 5–10. 10.1007/s11019-006-9017-3 16955344

[CR30] Paavola, Jouni. 2017. Health impacts of climate change and health and social inequalities in the UK. *Environmental Health* 16(1): 113. 10.1186/s12940-017-0328-z 29219089 10.1186/s12940-017-0328-z PMC5773866

[CR31] Page, Edward A. 2008. Distributing the burdens of climate change. *Environmental Politics* 17(4): 556–575. 10.1080/09644010802193419

[CR32] Parker, Joshua. 2022. Barriers to green inhaler prescribing: ethical issues in environmentally sustainable clinical practice. *Journal of Medical Ethics*, medethics-2022-108388. 10.1136/jme-2022-108388 10.1136/jme-2022-108388 PMC9887388 35981864

[CR33] Pellegrino, Edmund D. 2001. The Internal Morality of Clinical Medicine: A paradigm for the Ethics of the Helping and Healing professions. *Journal of Medicine and Philosophy* 26(6): 559–579. 10.1076/jmep.26.6.559.2998 11735050 10.1076/jmep.26.6.559.2998

[CR34] Persad, Govind, and Jessica du Toit. 2020. The case for valuing non-health and indirect benefits. In *Global Health Priority-Setting: Beyond cost-effectiveness*, ed. Ole F. Norheim, Ezekiel J. Emanuel, and Joseph Millum, 207–222.

[CR35] Peter, Fabienne. 2001. Health equity and social justice. *Journal of Applied Philosophy* 18(2): 159–170. 10.1111/1468-5930.00183 11785544

[CR36] Powers, Madison, and Ruth Faden. 2008. *Social justice: The moral foundations of public health and health policy*. Oup USA. 10.1007/s00103-008-0443-7 18259708

[CR37] Rao, Narasimha D., and Paul Baer. 2012. Decent living’ emissions: A conceptual Framework. *Sustainability* 4(4): 656–681. 10.3390/su4040656

[CR38] Rogelj, Joeri, Michiel Schaeffer, Pierre Friedlingstein, Nathan P. Gillett, P. Detlef, and van Vuuren. Keywan Riahi, Myles Allen, and Reto Knutti. 2016. Differences between carbon budget estimates unravelled. *Nature Climate Change* 6(3): 245–252. 10.1038/nclimate2868

[CR39] Salas, Renee N., Edward Maibach, David Pencheon, Nick Watts, and Howard Frumkin. 2020. A pathway to net zero emissions for healthcare. *BMJ*, 371. 10.1136/bmj.m3785 10.1136/bmj.m3785 33004403

[CR40] Schramme, Thomas. 2017. Goals of Medicine. In *Handbook of the philosophy of Medicine*, ed. Thomas Schramme, and Steven Edwards. 121–128. Dordrecht: Springer Netherlands. 10.1007/978-94-017-8688-1_5

[CR41] Segall, Shlomi. 2007. Is health care (Still) Special?*. *Journal of Political Philosophy* 15(3): 342–361. 10.1111/j.1467-9760.2007.00284.x

[CR42] Segall, Shlomi. 2010. Is health (Really) special? Health policy between rawlsian and luck egalitarian justice. *Journal of Applied Philosophy* 27(4): 344–358.

[CR43] Shue, Henry. 1993. Subsistence emissions and luxury emissions. *Law and Policy* 15(1): 39–59.

[CR44] Shue, Henry. 2014. *Climate justice: Vulnerability and protection*. Oxford University Press.

[CR45] Smiley, Marion. 2023. Collective responsibility. In *The Stanford Encyclopedia of Philosophy*, ed. Edward N. Zalta and Uri Nodelman, Fall 2023. Metaphysics Research Lab, Stanford University. https://plato.stanford.edu/archives/fall2023/entries/collective-responsibility/

[CR46] Tennison, Imogen, Sonia Roschnik, Ben Ashby, Richard Boyd, Ian Hamilton, Tadj Oreszczyn, Anne Owen, Marina Romanello, Paul Ruyssevelt, and Jodi Sherman et al. 2021. Health Care’s response to Climate Change: A Carbon Footprint Assessment of the NHS in England. *The Lancet Planetary Health* 5(February): e84–e92. 10.1016/S2542-5196(20)30271-0 33581070 10.1016/S2542-5196(20)30271-0 PMC7887664

[CR47] Vanderheiden, Steve. 2008. *Atmospheric justice: A political theory of climate change*. Oxford University Press.

[CR48] Venkatapuram, Sridhar. 2011. *Health justice: An argument from the capabilities approach*. Polity.

[CR49] Walzer, Michael. 1983. *Spheres of justice: A defence of pluralism and equality*. Basic Books.

[CR50] Watts, Nick, W. Neil Adger, Paolo Agnolucci, Jason Blackstock, Peter Byass, Wenjia Cai, Sarah Chaytor, Tim Colbourn, Mat Collins, Adam Cooper, et al. 2015. Health and climate change: Policy responses to protect public health. *Lancet (London England)* 386(10006): 1861–1914. 10.1016/S0140-6736(15)60854-6 26111439 10.1016/S0140-6736(15)60854-6

[CR51] Welton, Shelley. 2022. Neutralizing the atmosphere. *Yale Law Journal* 132(1): 171–249. https://www.yalelawjournal.org/feature/neutralizing-the-atmosphere

[CR52] Wiggins, David. 1987. Claims of need. In *Needs, values, Truth: Essays in the philosophy of Value*, Oxford: Oxford University Press.

[CR53] Wilkinson, Alex, and Ashley Woodcock. 2022. The Environmental Impact of inhalers for Asthma: A Green Challenge and a Golden Opportunity. *British Journal of Clinical Pharmacology* 88(7): 3016–3022. 10.1111/bcp.15135 34719810 10.1111/bcp.15135

[CR54] Wilson, J. 2009. Not so special after all? Daniels and the Social Determinants of Health. *Journal of Medical Ethics* 35(1): 3–6. 19103934 10.1136/jme.2008.024406

[CR55] World Health Organisation. 2023. Climate change. https://www.who.int/health-topics/climate-change

[CR56] World Health Organisation. 2024. Commitments to climate change and health. https://www.who.int/initiatives/alliance-for-transformative-action-on-climate-and-health/commitments

